# Has global deforestation accelerated due to the COVID-19 pandemic?

**DOI:** 10.1007/s11676-022-01561-7

**Published:** 2022-11-16

**Authors:** Jonnathan Céspedes, Janelle M. Sylvester, Lisset Pérez-Marulanda, Paula Paz-Garcia, Louis Reymondin, Mehran Khodadadi, Jhon J. Tello, Augusto Castro-Nunez

**Affiliations:** 1 The Alliance of Bioversity International and International Center for Tropical Agriculture (CIAT), Km 17 Recta Cali-Palmira, Cali, Colombia; 2 Department of Geosciences and Natural Resource Management, University of Copenhagen, Copenhagen, Denmark; 3 Leibniz Centre for Agricultural Landscape Research (ZALF), Müncheberg, Germany

**Keywords:** Deforestation, COVID-19, Time series, Terra-i, Drivers of deforestation monitoring

## Abstract

As the COVID-19 pandemic unfolded, questions arose as to whether the pandemic would amplify or pacify tropical deforestation. Early reports warned of increased deforestation rates; however, these studies were limited to a few months in 2020 or to selected regions. To better understand how the pandemic influenced tropical deforestation globally, this study used historical deforestation data (2004–2019) from the Terra-i pantropical land cover change monitoring system to project expected deforestation trends for 2020, which were used to determine whether observed deforestation deviated from expected trajectories after the first COVID-19 cases were reported. Time series analyses were conducted at the regional level for the Americas, Africa and Asia and at the country level for Brazil, Colombia, Peru, the Democratic Republic of Congo and Indonesia. Our results suggest that the pandemic did not alter the course of deforestation trends in some countries (e.g., Brazil, Indonesia), while it did in others (e.g., Peru). We posit the importance of monitoring the long-term effects of the pandemic on deforestation trends as countries prioritize economic recovery in the aftermath of the pandemic.

**Supplementary Information:**

The online version contains supplementary material available at 10.1007/s11676-022-01561-7.

## Introduction

The international community has shown concern over the possible effects of the COVID-19 pandemic on forest cover. Early press reports indicated that the global deforestation rate increased during the start of the pandemic (DW News 2020; Mongabay 2020), and publications warned this was a result of reduced governance and control in forested regions due to mandatory confinement measures (de Andreazzi et al. 2020; Fox et al. 2020; Golar et al. 2020). Similarly, various international organizations warned at the start of the pandemic that COVID-19 would threaten rural livelihoods and wildlife and that confinement could lead to possible variations not only in food consumption patterns but in the production of consumer goods (CI 2020; FAO 2020; Winter 2020). Despite early warnings, the effects of the pandemic on forests are not yet clearly understood.

Early studies reflect a consensus that the global deforestation rate increased in early 2020 and that this was likely due to the COVID-19 pandemic. Most of these studies, however, report results from qualitative analysis based on stakeholder interviews (Fox et al. 2020; Golar et al. 2020). Studies using quantitative methods coincide in the use of real or near real-time monitoring systems based on the use of satellite imagery, such as Moderate Resolution Imaging Spectroradiometer (MODIS), Landsat and Sentinel (Amador-Jiménez et al. 2020; Brancalion et al. 2020). However, most of these studies were reported between February and May 2020, during the initial phase of global mandatory confinement.

Consistently, researchers argue that the pandemic may have long-lasting economic and social impacts that will likely have continued ramifications for forests (López-Feldman et al. 2020; Zahawi et al. 2020). For instance, a crisis like COVID-19 could lead to increased forest fires due to reduced monitoring power (López-Feldman et al. 2020). Furthermore, COVID-19-related disruptions in the production and trade of timber and non-timber forest products pose a significant threat to the livelihoods and industries that depend on this sector (FAO 2020). The production of prohibited timber products, such as charcoal, has been expected to increase throughout the COVID-19 crisis as illegal activities with rapid economic gains replace disrupted livelihoods rooted in legal activities (FAO 2020). Additionally, the increasing unemployment in cities during the pandemic has driven migration to rural areas, at least temporarily, as people seek income through economic activities that may include agricultural production in forest frontiers, resulting in increased pressure on forests.

Conversely, according to Kaimowitz and Wunder (2021) and Wunder et al. (2021), the effects of COVID-19 in the early phases could have facilitated a decline in deforestation due to the economic recession induced by the confinement period. This idea is based on a decline in commodity demand due to the economic uncertainty faced by consumers and a dramatic fall in commodity prices between February and March 2020. While these trends appear to be consistent with other variables evaluated in these studies, such as the exchange rate and employment rate, there remains a need to explore the spatial and temporal behavior of deforestation as a physical variable independent of the economic situation during this time.

Several of the early studies on the effects of the pandemic on tropical deforestation are based on stakeholder interviews. For example, de Andreazzi et al. (2020) compile the opinions of several Brazilian scientists who argue that the deforestation rate in Brazil increased in 2020 due to relaxation of environmental protection laws, disassembly of environmental institutions, attacks on conservation organizations and disregard for scientific evidence. Similarly, after performing telephone interviews with 60 farmers in two major regions in Nepal, Fox et al. (2020) suggest that deforestation increased due to agricultural expansion caused by a massive return of workers to rural farms as a result of COVID-19 restrictions. Through semi-structured interviews, Golar et al. (2020) conclude that deforestation and timber logging increased due to a greater demand for forest products by rural communities in Indonesia. Troëng et al. (2020) agree that greater urban-to-rural migration increased pressure on forest resources in the tropics and that the pandemic has exacerbated deforestation, land grabbing, illegal mining and animal poaching carried out by criminal groups and opportunistic actors. López-Feldman et al. (2020) argue that it is too early to conclude that the pandemic has caused an increase in deforestation; however, their literature review suggests potential negative effects on forests by the presence of illegal armed groups, expansion of illicit crops, illegal mining and land grabbing, especially in Colombia. Lastly, Zahawi et al. (2020), which presents commentary by subject matter experts, suggests a recession in restoration activities due to the high costs of reforestation.

Fewer studies have reported results from quantitative analyses. Amador-Jiménez et al. (2020) evaluated the impact of COVID-19 in Colombia using data from the Visible Infrared Imaging Radiometer Suite (VIIRS) and MODIS sensors to assess locations of forest fire hotspots during the country’s lockdown period. The study reveals an increase in forest fires correlated with the presence of illegal armed groups, which may be driving an increase in deforestation. A similar methodology was presented by Brancalion et al. (2020) using a multi-temporal analysis of Global Land Analysis & Discovery (GLAD) alerts (Hansen et al. 2016), revealing that tropical deforestation increased in the first month following government confinement measures. They posit one reason for this initial increase could be restricted legal enforcement abilities allowing for higher illegal deforestation activities. According to Winter and Shapiro (2020), approximately 645,000 ha of forest cover were lost in March 2020, with the greatest declines seen in Indonesia (~ 130,000 ha; nearly three times the value seen in March 2019), the Democratic Republic of Congo (~ 100,000 ha) and Brazil (~ 95,000 ha). In Brazil, from January to April, deforestation alerts over indigenous regions increased by 59% compared with the same period in 2019 (Greenpeace 2020).

Most of these studies are based on data published by Brazil’s deforestation monitoring and alert generation system, the National Institute for Space Research (INPE; Bir 2020; Mongabay 2020; Walz 2020; Winter 2020). Generally, these studies compare data produced by the INPE during 2020 to previous years ranging to 2008. INPE’s monitoring system is calibrated, however, to the local Brazilian geographic context, which implies that alerts may be better applied to specific territorial and spatial conditions. Therefore, in order to release fitted data at the global scale, alternative near-real time monitoring systems must be used such as Terra-i (Reymondin et al. 2012), Forest Monitoring for Action (FORMA; Hammer et al. 2014), Global Forest Change (GFC; Hansen et al. 2013), GLAD (Hansen et al. 2016) and Radar for Detecting Deforestation (RADD; Reiche et al. 2021).

This study aims to fill this research gap and contribute to the understanding of the effects of the COVID-19 pandemic on deforestation. Specifically, we attempt to answer whether pantropical deforestation trends changed during the first year of the pandemic. To this end, we use data from the Terra-i deforestation monitoring system to identify changes in deforestation trends from 2004 through 2020 at the pantropical scale following four steps: (1) a mean time series was created for each continent showing the mean deforestation trend across the tropics; (2) Discrete Fourier Transform (DFT) was applied to each series to determine if the curve is governed by cyclical behaviors and with what frequency these cycles occur; (3) the expected mean deforestation trend for 2020 for each continent was projected; and, (4) the 2020 projections were compared with the observed mean deforestation trends after the date on which active cases of COVID-19 began to be reported in each continent as well as in five key tropical countries: Brazil, Indonesia, the Democratic Republic of the Congo, Peru and Colombia. Finally, we discuss results and conclude in light of available reports on the effects of the pandemic on tropical deforestation.

## Materials and methods

This study uses reports of deforestation alerts generated by Terra-i (Reymondin et al. 2012). Based on this dataset, we (1) analyzed the historical behavior of deforestation from 2004 to 2020 at the global scale, grouping the data reported for the pantropical countries at the continental level for Africa, the Americas and Asia; (2) produced mean deforestation trends and projected trends for 2020 for each continent based on an analysis of temporal patterns looking at the frequency of cycles of repeated events and the intensity of the events in terms of deforested area; (3) identified changes in deforestation trends for 2020 by comparing projected deforestation trends with observed mean deforestation trends; and (4) repeated the same analyses at the country level for Brazil, Colombia, Peru, the Democratic Republic of the Congo, and Indonesia. Lastly, we explored how the use of different monitoring systems could influence our results by intercomparing deforestation trends generated by Terra-i, GFC and GLAD alerts.

### Land cover change detection system: Terra-i

Terra-i detects vegetation changes across the tropics in near real-time. This system monitors variations in vegetation cover resulting from either human activities or natural disturbances (Reymondin et al. 2012). As input, Terra-i uses the Normalized Difference Vegetation Index of MODIS, which has a temporal resolution of 16 days and spatial resolution of 250 m (Huete et al. 1999), the precipitation products of the Tropical Rainfall Measuring Mission (TRMM) and the Global Precipitation Measurement (GPM).

The Terra-i alerts indicate pixels where a loss of vegetation cover was detected. Each alert (released every 16 days) represents one pixel with an area of 6.25 ha, according to the spatial resolution of the MODIS sensor. The system is based on the premise that vegetation follows a predictable pattern of changes in vigor due to specific geographic and climatic conditions (Carlson and Ripley 1997). These patterns are detected using artificial intelligence techniques, such as Bayesian neural networks (Bishop 2006), which are trained to identify patterns of change in plant vigor for a given geographic location. The result of this training allows for the detection of areas where the vigor of the vegetation is suddenly modified beyond the normal distribution limits of the identified and previously classified values.

### Dynamic analysis of temporal patterns of deforestation

The Terra-i alerts between 2004 and 2020, expressed in units of hectares (ha), were used to examine the temporal variability of deforestation in the Americas, Africa and Asia. A mean time series was created for each continent showing the mean deforestation trend across the tropics. This analysis uses a statistical description of the annual deforestation variations and applies the Discrete Fourier Transform (DFT) to each series to determine if the curve is governed by cyclical behaviors and with what frequency these cycles occur (Wen et al. 2004; Roerink et al. 2000; Verhoef et al. 1996). The DFT was obtained using the SciPy v1.5.4. module of the free access software Python 3.7 (Virtanen et al. 2020).

The above-mentioned time series were used to project the expected mean deforestation trend for 2020 for each continent based on the historical cyclical patterns observed throughout the analysis period. The 2020 projections were then used to assess whether there are differences in observed mean deforestation trends after the date on which active cases of COVID-19 began to be reported globally. These projections were generated using the ‘forecast’ and ‘auto.arima’ functions of the free access software R v1.3.1093 (Hyndman et al. 2020; Hyndman and Khandakar 2008), and the results were computed based on 30, 64 and 43 countries belonging to the Americas, Africa and Asia, respectively (Table S1).

### Dynamic analysis of temporal patterns of deforestation in key countries

The methodology described above was used to evaluate the deforestation trends for selected countries in each continent. The countries were selected from the three major tropical forest zones—the Amazon, the Congo basin and Southeast Asia—due to the valuable ecosystem services these regions provide. The focal countries consist of Brazil, Colombia, Peru, the Democratic Republic of the Congo (DRC) and Indonesia. These countries were chosen because their territories contain a large area of tropical forests with high biodiversity value and historically have been affected by many direct and indirect drivers of deforestation, such as illegal timber extraction, lack of governance and illicit crops (Hansen et al. 2013; Coca Castro 2015; Heino et al. 2015). The countries evaluated in this case study are those whose high rates of deforestation can determine the trend of the deforestation curve at the continental level. We demonstrate this by repeating the analysis conducted for the Americas with the exclusion of Brazil.

### Differences between the results retrieved by deforestation monitoring systems

**Fig. 1:**
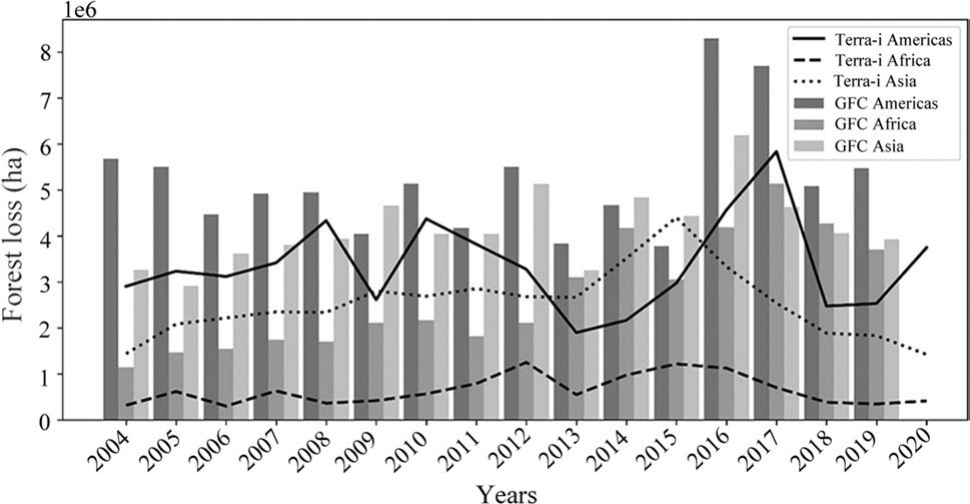
Historical (2004–2020) deforestation detected by the Terra-i and Global Forest Change (GFC) systems for the Americas, Africa and Asia

Lastly, we wanted to explore how the use of different deforestation monitoring systems could influence our results. The differences between time series generated using Terra-i (Reymondin et al. 2012) and GFC (Hansen et al. 2013, 2016) are shown in Fig. 1. The figure displays data from 2004 to 2020 for the Americas, Africa and Asia. Both systems have a global scale, but they have some visible key differences. For instance, we see that the Terra-i system, due to its 250 m spatial resolution, underestimates the observed deforestation values for each continent, while GFC with a spatial resolution of 30 m more accurately estimates deforestation given the level of pixel detail. Furthermore, at the time of analysis, GFC data was not available for 2020. Consequently, we can infer that actual deforestation rates could be higher than the rates expressed by Terra-i. This underestimation, however, is compensated by the availability of Terra-i’s uninterrupted temporal coverage from 2004 to 2020, with alerts released every 16 days. Despite these differences, Fig. 1 shows that the trend curves for each continent remain similar for both systems. This behavior indicates that regardless of the differences in detected deforested area, there are visible temporal patterns in each continent, which is key for evaluating the global deforestation rate.

**Fig. 2:**
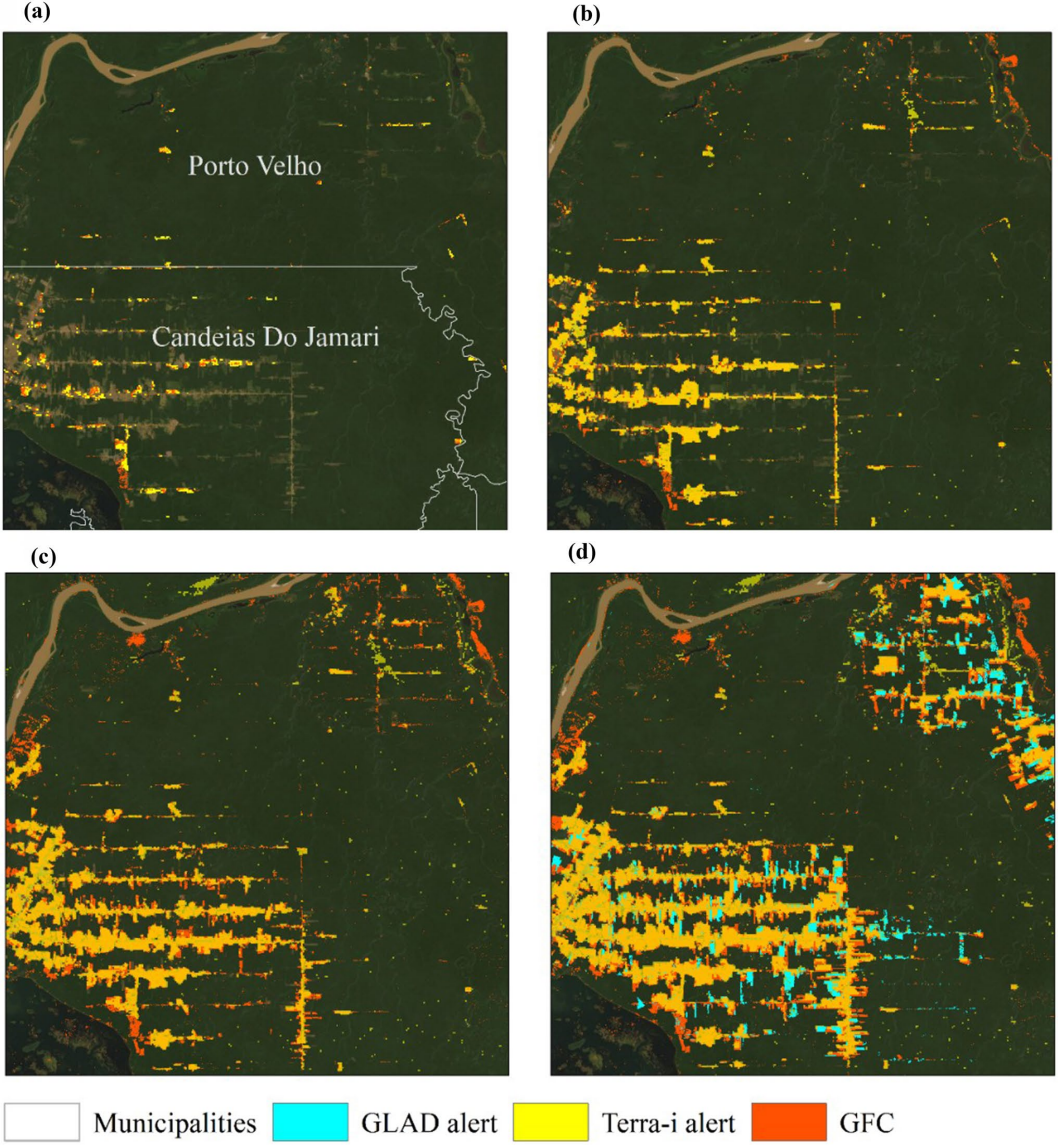
Deforestation in municipalities of Candeias do Jamari and Porto Velho in Rondonia State, Brazil. **a** Terra-i and Global Forest Change (GFC) data for 2004; **b** accumulated Terra-i and GFC data from 2004 to 2010; **c** Terra-i and GFC data from 2004 to 2016; and **d** Terra-i data from 2004 to 2020, GFC data from 2004 to 2019 and GLAD data for 2020

We further wanted to explore how this temporal behavior could be associated with the spatial distribution of deforestation. Figure 2 displays the spatial distribution of Terra-i, GFC and GLAD alert system detections in Rondonia State, Brazil. This figure shows how for 2004, 2010, 2016 and 2020, deforestation is concentrated in this location—with Terra-i detections again underestimating forest cover change. The GFC system can detect a greater area of deforestation than Terra-i due to its spatial resolution of 30 m; therefore, GFC is a useful monitoring system for detecting annual deforestation at the local and regional scale. However, despite the spatial resolution of Terra-i, Fig. 2 shows that this system is a powerful tool as its detections spatially match those of GFC. For the GLAD system, we only have data for 2020; however, we see that these detections also match those of Terra-i and GFC. Therefore, with this intercomparison, we conclude that the analysis proposed in this paper is capable of explaining the temporal and spatial patterns of deforestation and can effectively be used to better understand how the COVID-19 pandemic has influenced global deforestation.

## Results

### Changes in tropical deforestation trends between 2004 and 2020

**Fig. 3:**
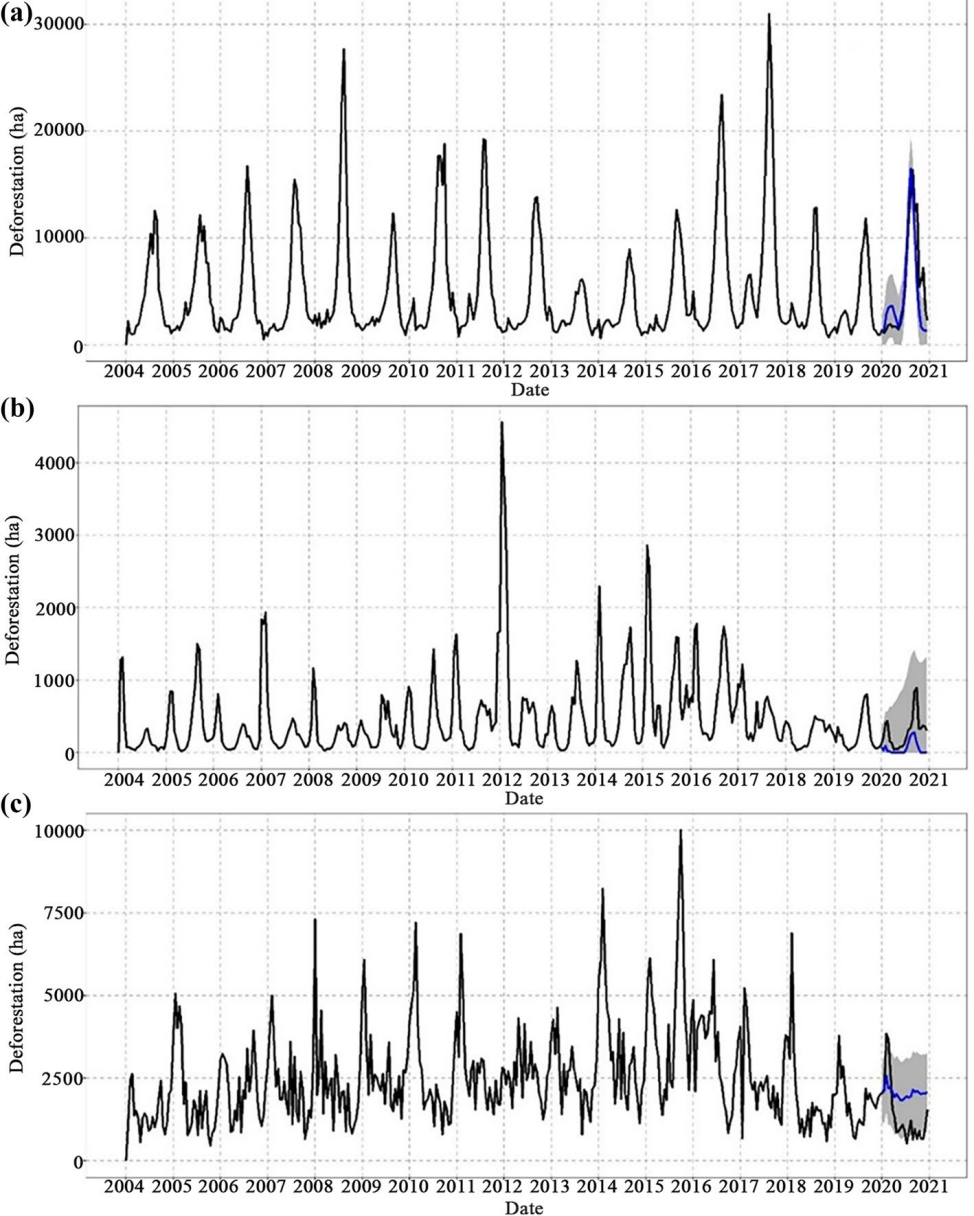
Time series of average deforestation in the pantropical zone of **a** the Americas, **b** Africa and **c** Asia. In each panel, the vertical dashed lines represent the beginning of a new calendar year. Each year consists of 23 Terra-i alert dates with a frequency of 16 days. The blue line represents the projection for 2020 based on the total time series. The shaded region represents the confidence interval of each projection

Figure 3 shows the trend of the time series and its projection in 2020 for the Americas, Africa and Asia. From these graphs, we evaluate the impact of the pandemic on deforestation by comparing the measured series of average deforestation for 2020 with the projection calculated for this year. The mean annual variation of deforestation across the study period is shown in Table 1. Most of the presented values for standard deviation are high because deforestation could reach zero ha many times each year.

**Table 1:** Annual variation of average deforestation for the Americas, Africa and Asia (mean ± SD) from 2004 to 2020

Region	2004	2005	2006	2007	2008	2009	2010	2011	2012
America	4212.6 ± 3707.8	4691.5 ± 3499.6	4522.6 ± 4560.9	4955.3 ± 4696	6284.1 ± 7313.1	3790.7 ± 3113.6	6340.5 ± 5836.9	5541 ± 5594.1	4750.6 ± 4272.1
Africa	235.9 ± 372	455.8 ± 454.9	222.9 ± 201.9	465 ± 609.3	269.1 ± 288.6	310.5 ± 227.5	419.1 ± 383	582.6 ± 497.3	922.7 ± 1275.3
Asia	1275 ± 530	1885 ± 1230.6	1987 ± 855.2	2140.2 ± 942.3	1963.5 ± 941	2403.4 ± 668.1	2390.4 ± 1282.6	2570 ± 958.9	2558.5 ± 844.4

Region	2013	2014	2015	2016	2017	2018	2019	2020
America	2753.9 ± 1648.7	3143.5 ± 2373.1	4313.5 ± 3687	6619.5 ± 6678.2	8460.7 ± 8385.6	3590.9 ± 3432.7	3671.8 ± 3225.1	5430.0 ± 4922.114
Africa	406.3 ± 364.3	720 ± 674.9	898.4 ± 766	831.4 ± 566.2	518.9 ± 314.6	284.1 ± 171.6	257.6 ± 243.7	245.1 ± 231.8
Asia	2463.3 ± 854	3271.9 ± 1603.9	4281.6 ± 2265.7	3193 ± 1438.5	2226.9 ± 865.4	1613.6 ± 903.4	1705 ± 667.1	1378.5 ± 923.1

*SD* standard deviation; Values reported in hectares

Data in Fig. 3a show that 2020 had the sixth highest peak in deforestation in the Americas since 2004 with higher values than those in 2019 and 2018. In contrast, annual deforestation in Africa (Fig. 3b) and Asia (Fig. 3c) decreased in 2020, reaching some of the lowest annual mean values in the time series. In the Americas, the deforestation series presents a wave behavior whose approximate period of oscillation is one year, reaching maximum peaks approximately every 0.5 years (Fig. 3a). That is, this average curve presents minimum values at the beginning of each year, grows to a maximum peak in the middle of the year and then descends to reach minimum values at the end of the year.

**Fig. 4:**
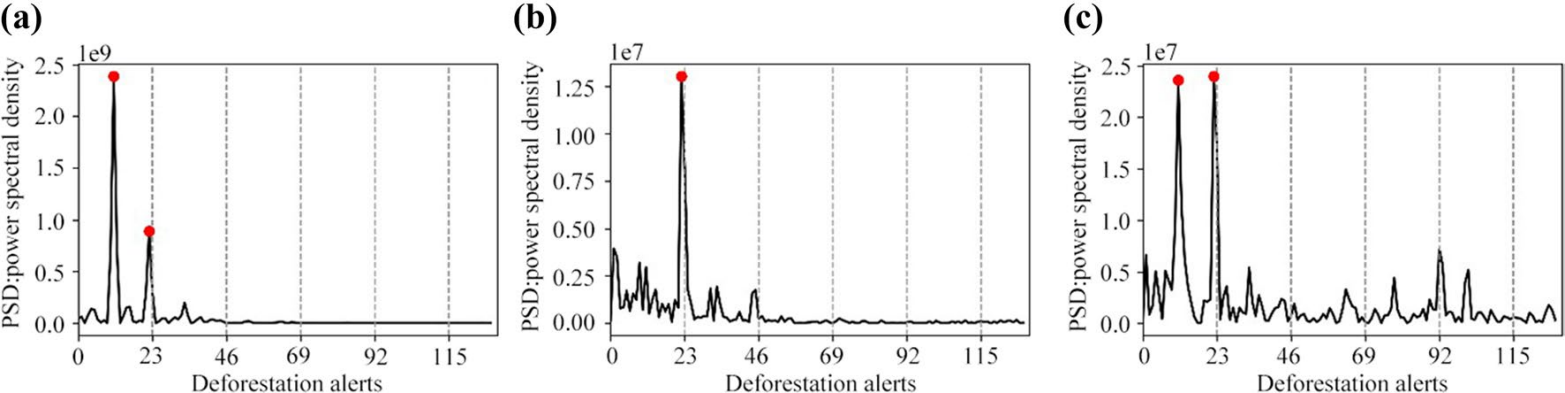
Time series of average deforestation in the frequency domain for **a** the Americas, **b** Africa and **c** Asia. The red dots indicate the apparent cycles that deforestation follows on each continent and the dashed lines indicate the number of Terra-i system updates per year

Figure 4a shows a breakdown of frequencies for the deforestation series in the Americas. Two peaks are observed, indicating that deforestation in the Americas is governed by two cycles: the first repeats approximately every six months and the second approximately every 12 months. The six-month cycle represents the growth of deforestation in the first half of the year, while the annual cycle includes the growth and decline of the curve. The six-month cycle is more intense because every first semester, deforestation reaches maximum peaks close to approximately 30,000 ha, while the intensity of the annual cycle is lower because it includes the processes of growth and decline in deforestation, which implies that the amplitude of the curve is less than the maximum peaks. This well-defined temporal pattern seen throughout the time series for the Americas is also observed in 2020. Figure 3a shows that this cyclical behavior is completed in full in 2020 and that the six-month cycle is more intense than in 2019 and 2018. Given these results, we conclude that the behavior of the deforestation trend in the Americas did not change during the first year of the COVID-19 pandemic.

Furthermore, as seen in Fig. 3a, the deforestation projection for 2020 predicts a maximum growth and a peak in the first six months, completing the first cycle of the year; consequently, the Terra-i observations reliably follow this prediction and the cyclical behavior. Thus, following the patterns observed in the historical series, the expected cycles took place in 2020. This year yielded the highest deforestation rate in the last three years of the presented history for the Americas with an average rate of 5430 ha.

Although the time series for Africa and Asia in Figs. 3b and 3c do not show a well-defined or periodic wave evolution like that of the Americas, the results of the DFT indicate that deforestation cycles do exist in these continents (Fig. 4). Figure 3b shows that in Africa, no temporal pattern can be identified as a period of oscillation of the series. Moreover, from 2010 to 2017, an increase is observed in the number of peaks per year, reaching up to three maximum values per period. Figure 4b shows that Africa exhibits only one high-intensity annual cycle, which indicates that this cycle involves the development of local maximums on the curve, particularly at the end of every year. Although only one cycle is defined, the spectral density exhibits a slight decrease between frequencies 0 and 23. This may indicate that in the average deforestation curve for Africa, slow transitions occur over the course of the year, which explains a single cycle.

Similarly, Fig. 3c shows that in Asia, no periodic oscillation pattern is observed. In contrast to Africa, deforestation in Asia follows two cycles similar to the Americas—one semi-annual and the other annual. Given that both cycles have a similarly high intensity, this implies that the level of deforestation in these cycles is similar. The local maximums representing these cycles are estimated to be approximately 5000 ha. The difference between the cycles in Asia and the Americas is that in Asia, deforestation undergoes an upward evolution from the last quarter of the year and reaches the local maximum at the beginning of each year; thus, the behavior observed for 2020 in Fig. 3c can be considered historically conventional, since a decreasing behavior is observed.

### Changes in deforestation trends between 2004 and 2020 in selected countries

**Fig. 5:**
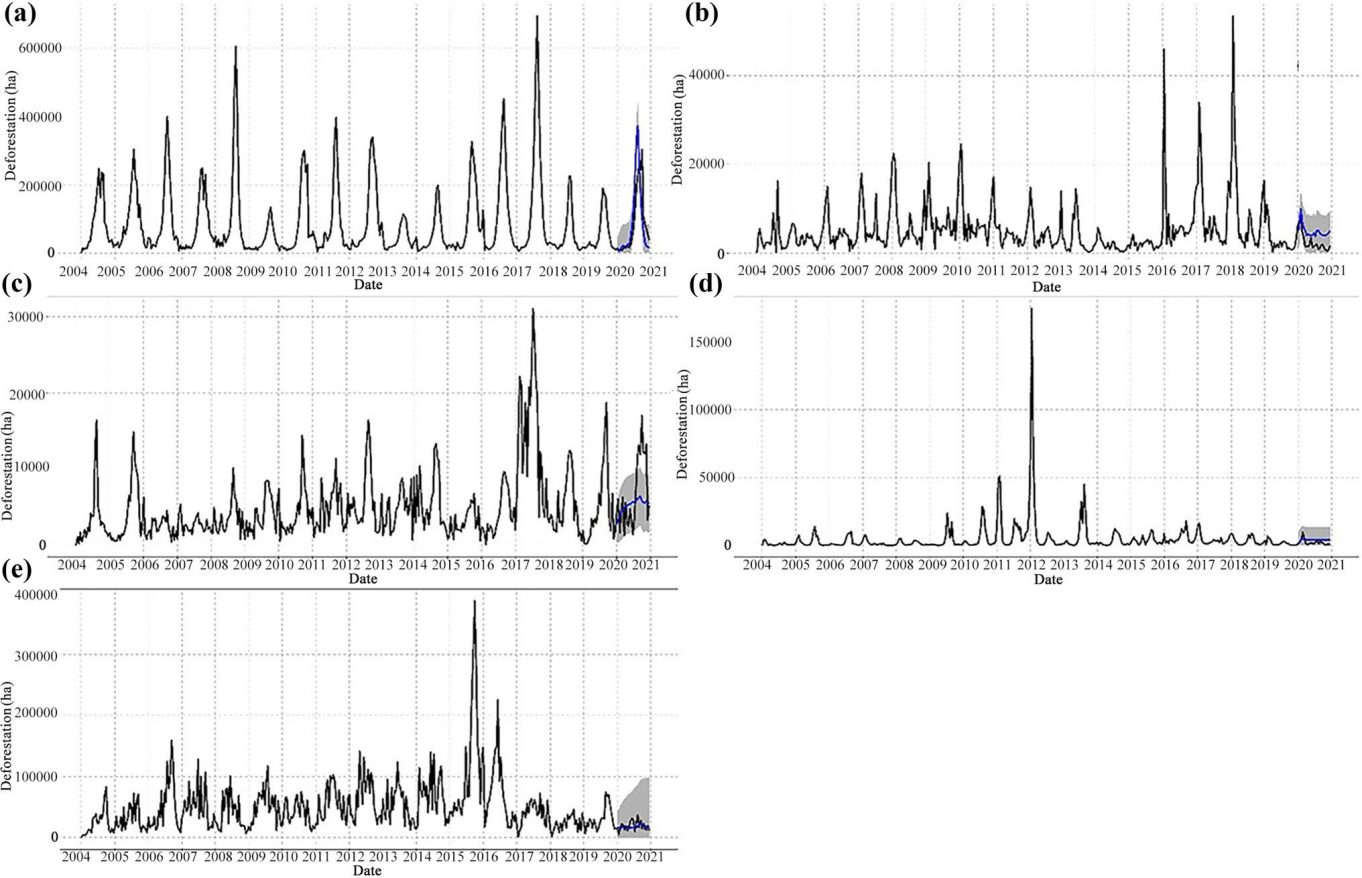
Deforestation projections for 2020 in **a** Brazil, **b** Colombia, **c** Peru, **d** the Democratic Republic of the Congo and **e** Indonesia. The projections have been prepared based on the 2004–2019 period for each country. The blue line represents the projection for 2020 based on the total of the series. The shaded region represents the confidence interval of each projection

The time series of average tropical deforestation from 2004 to 2019 for Brazil, Colombia, Peru, the DRC and Indonesia are presented in Fig. 5. The graphs display the actual deforestation curve for each country and a projection of deforestation for 2020 that is useful for identifying whether there have been sudden changes in deforestation patterns.

Figure 5a shows Brazil’s historical deforestation series and projection for 2020 based on the time series. The projected 2020 deforestation trend for Brazil indicates a strong, accelerated growth trend in the first half of the year, similar to the time series of the Americas. The observed data are consistent with its projection; however, the actual curve is slightly below the projection curve. This behavior points out that deforestation in Brazil for the analyzed 2020 period has followed an expected pattern. Likewise, a turning point is observed with a maximum of approximately 300,000 ha and a decline occurring after August 28, as in the series of the Americas. Results show that deforestation in Brazil has reached levels higher than 2018 and 2019, but lower than expected, according to the numerical projection.

Figure 5b, c show that deforestation in Colombia and Peru behaved differently from what was expected based on projections. In the case of Colombia, there was a peak of approximately 10,000 ha in the first two months of the year that subsequently decreases. Although the observed trend follows the same projected peak, the expected value was higher, close to 15,000 ha. Despite the peak between January and February, the actual deforestation trend shifted toward a decline. The Colombian deforestation curve for 2020 is similar in intensity and geometry to that reported in 2019; during both years, the maximum peak was located approximately between January and February with an average value of 18,000 ha.

In the case of Peru, deforestation shows an increasing trend (Fig. 5c). For 2020, the deforestation observed was much higher than predicted by the projection, reaching one of the highest peaks of historical deforestation at approximately 18,000 ha. Moreover, in addition to this 2020 peak, deforestation in Peru reached roughly 14,000 ha in the last six months of the year.

Figure 5d shows that in the case of the Democratic Republic of the Congo, the observed deforestation had a maximum peak of approximately 13,000 ha at the end of February before registering a declining behavior. Contrary to this, the projection presents no peak but does exhibit a trend higher than the curve of observed values.

In Indonesia, the observed deforestation curve presents a more volatile behavior than the calculated projection; the maximum and minimum peaks of the observed curve are more extreme, but the average of the observed trend adjusts to the average trend of the projection, which presents a much more stable behavior with marginal differences between the ends of the curve (Fig. 5e). In both cases, the average trend is around 20,000 ha. Thus, it can be said that for Indonesia there is no striking divergence between what is observed and what is expected.

**Fig. 6:**
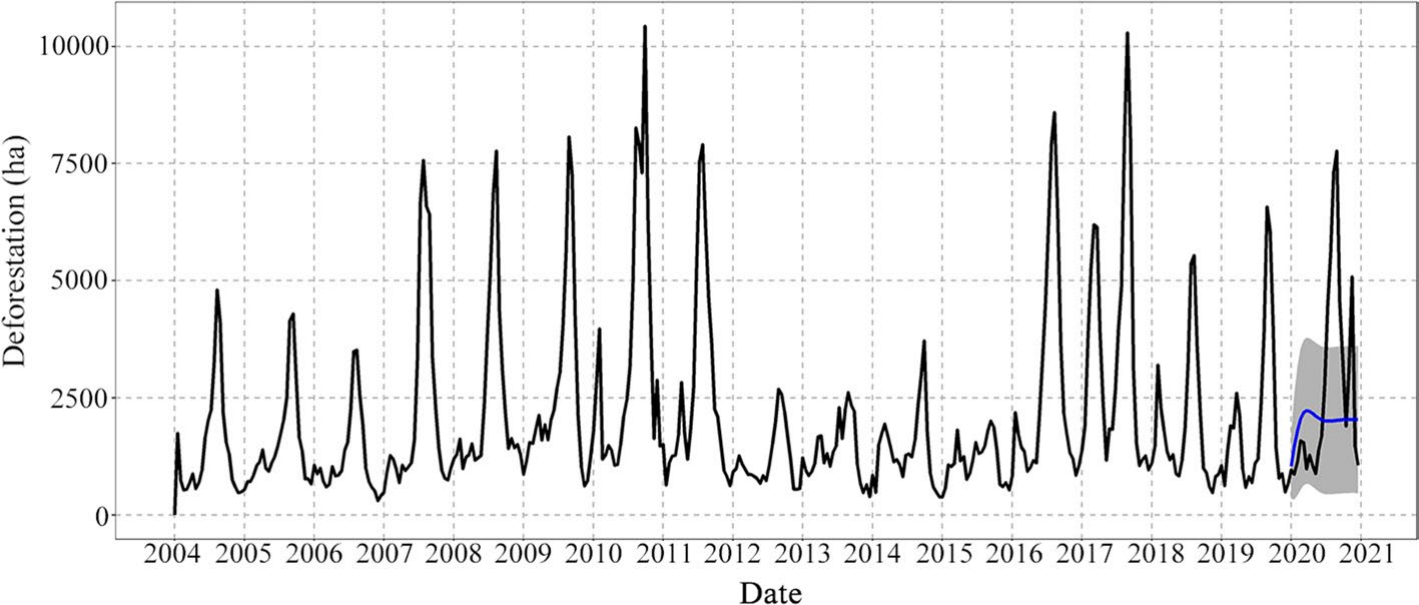
Time series and numerical projection of average deforestation in the Americas, excluding Brazilian deforestation

Figure 6 shows an average deforestation scenario excluding the alerts detected in Brazil. We again report both the time series of deforestation and the numerical projection based on the historical series. This scenario displays lower deforestation levels than Fig. 3a—nearly a third of what was observed with full data—and shows that the numerical projection has changed. This new projection displays an average curve between maximum and minimum values but does not keep the original geometry following the two-cycle behavior previously discussed.

## Discussion

The results of this study reveal that during the first year of the COVID-19 pandemic, deforestation in the Americas and Asia did not exceed projected trends for 2020 based on historical data. Conversely, a marginal increase was observed for Africa with values rising just above 2019 levels but remaining generally low compared to the values of the Americas and Asia. Several early studies and media reports comparing deforestation alerts during the onset of pandemic to the same months in previous years suggested that the pandemic was fueling an increase in tropical deforestation rates (Brancalion et al. 2020; Taylor 2020; Winter 2020). However, such a comparison does not necessarily reflect long-term trends and fluctuations in deforestation at the regional or national level. By incorporating historical deforestation data (2004–2020) and analyzing cyclical patterns of deforestation in these regions, this study was able to assess whether observed deforestation trends in 2020 deviated from what was expected for the year in the pantropical regions.

Studies referencing GLAD data documented disconcerting increases in deforestation alerts in 2020 compared to previous years, generally ascribing these increases to reduced forest governance and increased land grabbing activities following national lockdown measures (Brancalion et al. 2020; Winter 2020). Other media sources relying mostly on site-specific reports, interviews, anecdotal accounts or limited satellite data also posited a direct relationship between increasing deforestation rates and the development of the pandemic (DW News 2020; Fair 2020; Taylor 2020; Unearthed 2020). However, several sources argue that deforestation in many areas was already on the rise at the start of 2020 before lockdown measures went into place (Fair 2020; López-Feldman et al. 2020; Saavedra 2020; Wunder et al. 2021).

### Effects of the COVID-19 pandemic on deforestation in the Americas

Our results suggest that the increasing deforestation trend observed in the Americas was expected for the year and that at the regional level the pandemic did not decisively change the trajectory of forest cover loss. These results are supported by Saavedra (2020) who found that increases in deforestation alerts in primary forests across the tropics were attributed to already increasing deforestation trends that preceded the pandemic and not to the lockdown measures implemented in the different countries. He reports an increase of 150,000 bi-weekly deforestation alerts from 2019 to 2020, but generally no statistically significant difference in deforestation alerts before and after each country imposed lockdown measures. He does, however, report a differential increase in alerts in Mexico and Brazil and a differential decrease in Colombia.

Similarly, we found that deforestation in Colombia fell below the predicted trend for 2020. Initially in the year, deforestation in Colombia exhibited an upward trend, following a similar cycle as the years before. This initial spike in the year, which is commonly observed throughout the time series, likely explains the significant differential decrease in deforestation found by Saavedra (2020). While deforestation in Colombia was lower than expected according to our projection, the country still registered an increase from 2019 to 2020 after experiencing a decline in deforestation from 2018 to 2019 (IDEAM 2021; Weisse and Goldman 2021). Conversely, as shown in the time series, deforestation in Brazil annually peaks in or after the middle of the year, corresponding to the dry and burning season beginning in late June (Moutinho et al. 2020), which could explain the differential increase found by Saavedra (2020). While our results confirm a stark increase in Brazilian deforestation alerts from 2019 to 2020, as reported by others (Daly 2020; Fair 2020; Weisse and Goldman 2021; Wunder et al. 2021), we found that this increase aligned with our projection. That is, based on the historical data, this increase in deforestation was expected for the year. Therefore, we argue that the observed rise in Brazilian deforestation was likely not attributable to the COVID-19 pandemic.

In contrast, deforestation in Peru exhibited a considerable spike in the second half of 2020 that well exceeded the predicted trend. The country historically has been subjected to high rates of illegal timber harvesting, and Weisse and Goldman (2021) report the appearance of new logging roads throughout the Peruvian Amazon in 2020. Anecdotal accounts also emerged from Peru citing observed increases in illegal logging and mining activities in the region throughout the year (Fair 2020; Dil et al. 2021). In May 2020, the government announced that its four-phase economic reopening plan would commence with the forestry, mining and hydrocarbon sectors. Simultaneously, environmental monitoring reports were suspended and extractive activities were promoted to encourage economic recovery (OECD 2020; Dil et al. 2021; Dummett et al. 2021). Because Peruvian deforestation characteristically peaks later in the year as seen in the time series, studies examining deforestation before and after the start of lockdown are unlikely to register a relationship between deforestation and pandemic-related restrictions, at least initially. Thus, more studies are needed to closely examine the short- to long-term impacts of the pandemic and associated economic recovery efforts on forest cover in Peru.

Our results from the Americas demonstrate that Brazil’s deforestation curve strongly influences the average deforestation curve observed at the continental level—both in terms of hectares and geometrical behavior of the curve. The similarities between the Brazilian deforestation curve and that of the Americas at the continental level reflect the significant contribution Brazil makes to deforestation in this region. With the exclusion of Brazil, we found that deforestation in this region surpassed what was projected for the year, possibly reflecting the increase seen in Peru or in other countries in the region. For example, Weisse and Goldman (2021) found that Bolivia had the third largest loss of primary forest area in 2020.

### Effects of the COVID-19 pandemic on deforestation in Africa

Deforestation in Africa rose slightly above the expected trend for the year, suggesting that some pandemic-related factors and restrictions may have influenced forest cover changes in the region. Across Sub-Saharan Africa, Kganyago and Shikwambana (2021) found that biomass burning emissions increased during the COVID-19 lockdown period, resulting mostly from the burning of forests, shrublands and cultivated lands. The authors found a higher density of fires in 2020 compared to 2019, with fires being concentrated mainly in the DRC and Angola during the initial lockdown periods (27 March–31 May). They attribute this increase in fires to meteorological and vegetation conditions that may have favored the spread of fires from agricultural activities and to the closure of wildfire management and control agencies during the lockdown periods. It is possible that local-scale forest cover loss resulting from an increase in fires in 2020 is underrepresented in this study given the 250 m spatial resolution of the Terra-i system. Furthermore, deforestation in tropical Africa historically has been driven by small-scale shifting agriculture and wood energy demands (Weisse and Goldman 2021), which also may be underrepresented at this resolution.

The effects of the pandemic on deforestation in this region may be prolonged given that economic recovery in Sub-Saharan Africa is expected to be slow. The International Monetary Fund (IMF) predicts the region will have the world’s slowest economic growth in 2021, with per capita GDP in many of the region’s countries not returning to pre-crisis levels until late 2025 (Selassie and Hakobyan 2021). Notably, post-pandemic economic recovery in the DRC will rely heavily on the mining sector, as the Congolese economy is driven mainly by the extraction of mineral resources. As of 2019, the sale of mining and hydrocarbon products abroad accounted for more than 90% of the country's export revenues (Modeawi et al. 2021). Furthermore, Dil et al. (2021) report of government rollbacks on environmental safeguards in the DRC in July 2020 in order to facilitate large-scale mining.

### Effects of the COVID-19 pandemic on deforestation in Asia

At the regional level in Asia, deforestation fell below the predicted 2020 trajectory and a declining deforestation trend was observed across recent years. The time-series projection for Indonesia revealed a similar decline in 2020 with observed deforestation rates generally following the expected trajectory. These results thus suggest that the declining patterns seen in these regions in previous years continued into 2020. Our findings are corroborated by recently released GFC data (Hansen et al. 2013) and Terra-i data showing a consecutive decline in tree cover loss over the last four years (2016–2020) in Indonesia (Global Forest Watch 2021). On the other hand, these results contradict early reports on GLAD alerts warning that deforestation was on the rise in the region (Brancalion et al. 2020; Winter 2020). For example, Brancalion et al. (2020) found a 63% increase in deforestation relative to 2019 in Asia–Pacific in the first month after local confinement measures were implemented. Winter (2020) reported that deforestation in Indonesia was up 130% compared to the March average of 2017–2019.

There are reasonable explanations for these discrepancies. As discussed in Weisse and Pickens (2020) and Wunder et al. (2021), GLAD alerts are useful for identifying recent possible areas of concern; however, certain limitations make them unsuitable for trend assessments over time—namely a six-month long confirmation process, confirmation complications due to cloud cover and inaccuracies due to false positives (Weisse and Pickens 2020). Therefore, a number of initially detected alerts may eventually be removed if they cannot be confirmed. Furthermore, month-to-month comparisons between years do not reflect variable fluctuations in deforestation that may differ among years, as seen in our time series for regional Asia and Indonesia, which may provide a false impression of directional trends.

While deforestation in Indonesia appears to be on a favorable downward trajectory, the question remains as to how the mid- and long-term economic effects of the pandemic may impact future deforestation rates. For example, in response to the pandemic-induced recession, the Indonesian government passed the Omnibus Law on Job Creation in October 2020 to promote economic growth and expand industrial development in the country. This law, however, also weakened environmental protection laws, modified procedures for land use change and placed state ownership over untitled lands (Dil et al. 2021; Weisse and Goldman 2021).

### Future risks for forests

While studies from 2020 focused on the initial impacts of the pandemic and associated confinement measures on deforestation rates, concerns should now be shifted toward national economic recovery plans and the short- to long-term impacts they may have on forest cover. Developing countries in the tropics face considerable economic fallout from the pandemic. Among the three pantropical regions, the Latin America and the Caribbean region was hardest hit, seeing a 7% decline in GDP, compared with Sub-Saharan Africa (− 1.9%) and the Association of Southeast Asian Nations (− 3.4%; IMF 2021; Wunder et al. 2021). For developing nations in these regions, addressing the consequential economic crisis will be a significant challenge given that these nations tend to have higher levels of debt, reduced borrowing capacity and fewer financial resources for economic stimulus packages compared with developed nations (Wunder et al. 2021).

Such economic challenges will likely impact the availability of government financial resources for sectors such as agriculture, protected areas and forest governance enforcement (Wunder et al. 2021). Furthermore, these pandemic-induced recessions may provide impetus to expand extractive activities and place more pressure on natural resources. For example, Peru experienced an 11.1% contraction in GDP in 2020—worse than that of Colombia (− 6.8%) and Brazil (− 4.1%; IMF 2021). Peru’s economy relies heavily on mining and hydrocarbon extraction, with mining accounting for 10% of GDP and 60% of exports (Dil et al. 2021). Deforestation in Peru also exceeded the predicted trajectory for 2020 based on our time series analysis. Consequentially, for nations that rely heavily on extractive activities, there is a risk that economic recovery may come at a high environmental cost.

## Conclusions

This study provides a unique perspective on deforestation during the initial year of the COVID-19 pandemic by comparing observed pantropical deforestation trends to projected trajectories based on historical data from 2004 to 2019. At the continental level, deforestation in the Americas followed the projected trend for 2020, while deforestation in Asia fell below and deforestation in Africa exceeded the projected trajectory. Deforestation at the continental level in the Americas, however, was highly influenced by deforestation in Brazil; with Brazil excluded, deforestation surpassed the 2020 projection. At the national level, Peru was the only country where deforestation strongly surpassed the trend predicted for the year. Observed increases in deforestation in Brazil aligned with our projection, suggesting the pandemic did not markedly change the course of deforestation in the country. Lastly, this study provides an opportunity to examine how the use of different deforestation monitoring systems may influence reporting, highlighting the utility of trend assessments when comparing deforestation data between years. While deforestation in the initial year of the pandemic appears to have followed pre-existing trends in several countries, we warn that pandemic-related national economic recovery efforts will continue to present risks for forest cover.

## Supplementary Information

Below is the link to the electronic supplementary material.

Supplementary file1 (DOCX 19 KB)
