# Supplementary material for: Has global deforestation accelerated due to the COVID-19 pandemic?
Source: J For Res (Harbin). 2022 Nov 16:1–13. Online ahead of print. doi: 10.1007/s11676-022-01561-7 (PMC9666988; doi:10.1007/s11676-022-01561-7)
Supplement: Supplementary file 1 — Supplementary file1 (DOCX 19 KB) [file 11676_2022_1561_MOESM1_ESM.docx]

**Table S1.** List of countries included in the analysis. Pantropic-level results were computed based on 137 countries belonging to the pantropic. Continental-level results were computed based on 30 countries belonging to the Americas, 64 belonging to Africa and 43 belonging to Asia.

| **id** |  | **Continent** | **Country** |
| --- | --- | --- | --- |
| 12 |  | America | Argentina |
| 28 |  | America | Belize |
| 33 |  | America | Bolivia |
| 37 |  | America | Brazil |
| 51 |  | America | Chile |
| 57 |  | America | Colombia |
| 61 |  | America | Costa Rica |
| 63 |  | America | Cuba |
| 72 |  | America | Dominican Republic |
| 73 |  | America | Ecuador |
| 75 |  | America | El Salvador |
| 86 |  | America | French Guiana |
| 99 |  | America | Grenada |
| 100 |  | America | Guadeloupe |
| 103 |  | America | Guatemala |
| 107 |  | America | Guyana |
| 108 |  | America | Haiti |
| 111 |  | America | Honduras |
| 123 |  | America | Jamaica |
| 162 |  | America | Mexico |
| 180 |  | America | Nicaragua |
| 191 |  | America | Panama |
| 194 |  | America | Paraguay |
| 195 |  | America | Peru |
| 200 |  | America | Puerto Rico |
| 211 |  | America | Saint Vincent and the Grenadines |
| 233 |  | America | Suriname |
| 246 |  | America | Trinidad and Tobago |
| 260 |  | America | Uruguay |
| 263 |  | America | Venezuela |
| 4 |  | Africa | Algeria |
| 6 |  | Africa | Sudan |
| 8 |  | Africa | Angola |
| 29 |  | Africa | Benin |
| 35 |  | Africa | Botswana |
| 42 |  | Africa | Burkina Faso |
| 43 |  | Africa | Burundi |
| 45 |  | Africa | Cameroon |
| 47 |  | Africa | Cape Verde |
| 47 |  | Africa | Cape Verde |
| 49 |  | Africa | Central African Republic |
| 50 |  | Africa | Chad |
| 58 |  | Africa | Comoros |
| 59 |  | Africa | Congo |
| 66 |  | Africa | Cote dIvoire |
| 68 |  | Africa | Democratic Republic of the Congo |
| 70 |  | Africa | Djibouti |
| 74 |  | Africa | South Sudan |
| 76 |  | Africa | Equatorial Guinea |
| 77 |  | Africa | Eritrea |
| 79 |  | Africa | Ethiopia |
| 89 |  | Africa | Gabon |
| 90 |  | Africa | Gambia |
| 90 |  | Africa | Gambia |
| 91 |  | Africa | Gaza Strip |
| 91 |  | Asia | Gaza Strip |
| 94 |  | Africa | Ghana |
| 105 |  | Africa | Guinea-Bissau |
| 106 |  | Africa | Guinea |
| 121 |  | Africa | Israel |
| 121 |  | Asia | Israel |
| 133 |  | Africa | Kenya |
| 142 |  | Africa | Lesotho |
| 144 |  | Africa | Liberia |
| 145 |  | Africa | Libya |
| 150 |  | Africa | Madagascar |
| 152 |  | Africa | Malawi |
| 155 |  | Africa | Mali |
| 159 |  | Africa | Mauritania |
| 160 |  | Africa | Mauritius |
| 161 |  | Africa | Mayotte |
| 169 |  | Africa | Morocco |
| 170 |  | Africa | Mozambique |
| 172 |  | Africa | Namibia |
| 181 |  | Africa | Niger |
| 182 |  | Africa | Nigeria |
| 205 |  | Africa | Rwanda |
| 206 |  | Africa | Reunion |
| 214 |  | Africa | Sao Tome and Principe |
| 217 |  | Africa | Senegal |
| 220 |  | Africa | Seychelles |
| 221 |  | Africa | Sierra Leone |
| 226 |  | Africa | Somalia |
| 227 |  | Africa | South Africa |
| 229 |  | Africa | Spain |
| 235 |  | Africa | Swaziland |
| 243 |  | Africa | Togo |
| 248 |  | Africa | Tunisia |
| 253 |  | Africa | Uganda |
| 257 |  | Africa | United Republic of Tanzania |
| 268 |  | Africa | Western Sahara |
| 270 |  | Africa | Zambia |
| 271 |  | Africa | Zimbabwe |
| 40765 |  | Africa | Egypt |
| 1 |  | Asia | Afghanistan |
| 13 |  | Asia | Armenia |
| 19 |  | Asia | Azerbaijan |
| 21 |  | Asia | Bahrain |
| 23 |  | Asia | Bangladesh |
| 31 |  | Asia | Bhutan |
| 44 |  | Asia | Cambodia |
| 115 |  | Asia | India |
| 116 |  | Asia | Indonesia |
| 117 |  | Asia | Iran (Islamic Republic of) |
| 118 |  | Asia | Iraq |
| 126 |  | Asia | Japan |
| 130 |  | Asia | Jordan |
| 137 |  | Asia | Kuwait |
| 138 |  | Asia | Kyrgyzstan |
| 139 |  | Asia | Lao People's Democratic Republic |
| 141 |  | Asia | Lebanon |
| 149 |  | Asia | Macau |
| 153 |  | Asia | Malaysia |
| 154 |  | Asia | Maldives |
| 175 |  | Asia | Nepal |
| 187 |  | Asia | Oman |
| 188 |  | Asia | Pakistan |
| 192 |  | Asia | Papua New Guinea |
| 196 |  | Asia | Philippines |
| 201 |  | Asia | Qatar |
| 202 |  | Asia | Republic of Korea |
| 202 |  | Asia | Republic of Korea |
| 215 |  | Asia | Saudi Arabia |
| 222 |  | Asia | Singapore |
| 231 |  | Asia | Sri Lanka |
| 239 |  | Asia | Tajikistan |
| 240 |  | Asia | Thailand |
| 242 |  | Asia | Timor-Leste |
| 249 |  | Asia | Turkey |
| 255 |  | Asia | United Arab Emirates |
| 261 |  | Asia | Uzbekistan |
| 264 |  | Asia | Viet Nam |
| 269 |  | Asia | Yemen |
| 33364 |  | Asia | Hong Kong |
| 147295 |  | Asia | China |
| 147295 |  | Asia | China |
| 147296 |  | Asia | Taiwan |
